# Supplementary figures and images for: Efficient replication of influenza D virus in the human airway underscores zoonotic potential
Source: bioRxiv. 2026 Feb 8:2026.02.07.704474. Preprint. [Version 1] doi: 10.64898/2026.02.07.704474 (PMC12889682; doi:10.64898/2026.02.07.704474)

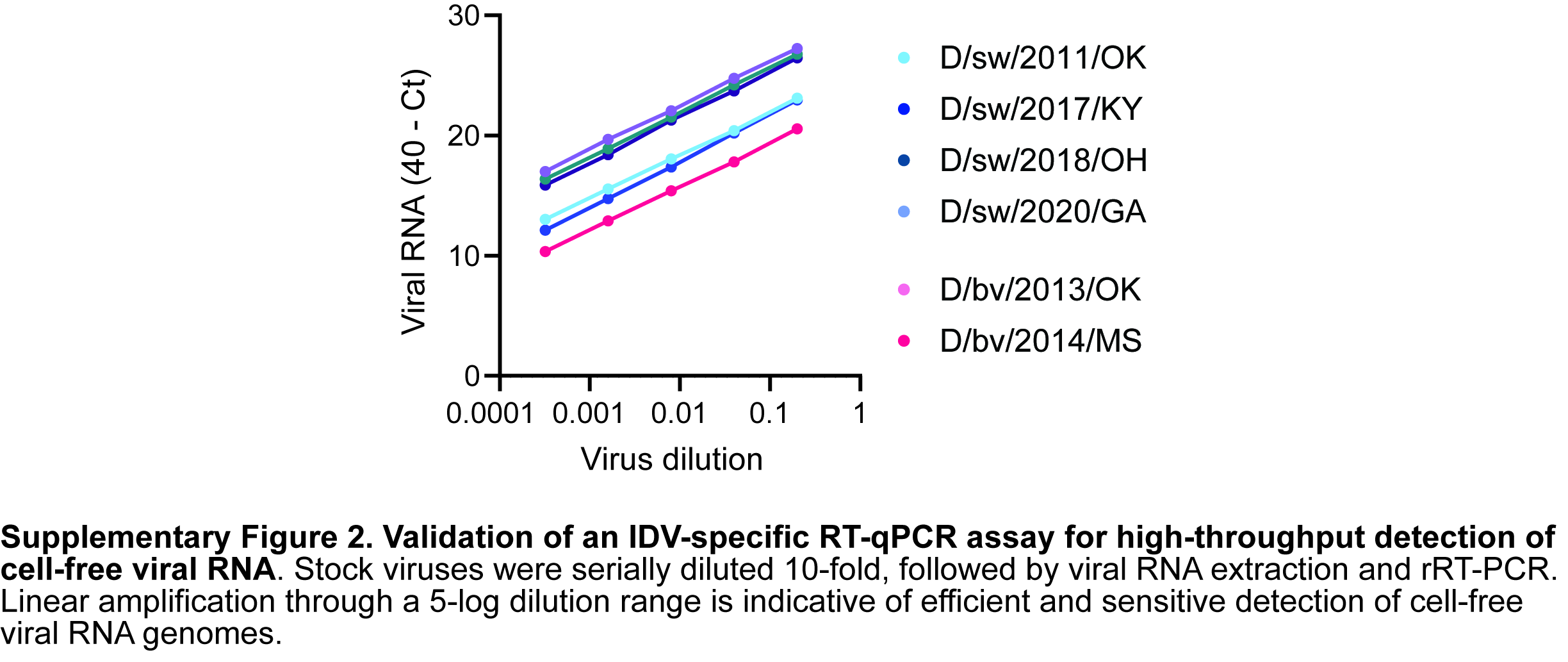

Supplement: Supplement 1 [file media-1.tif]

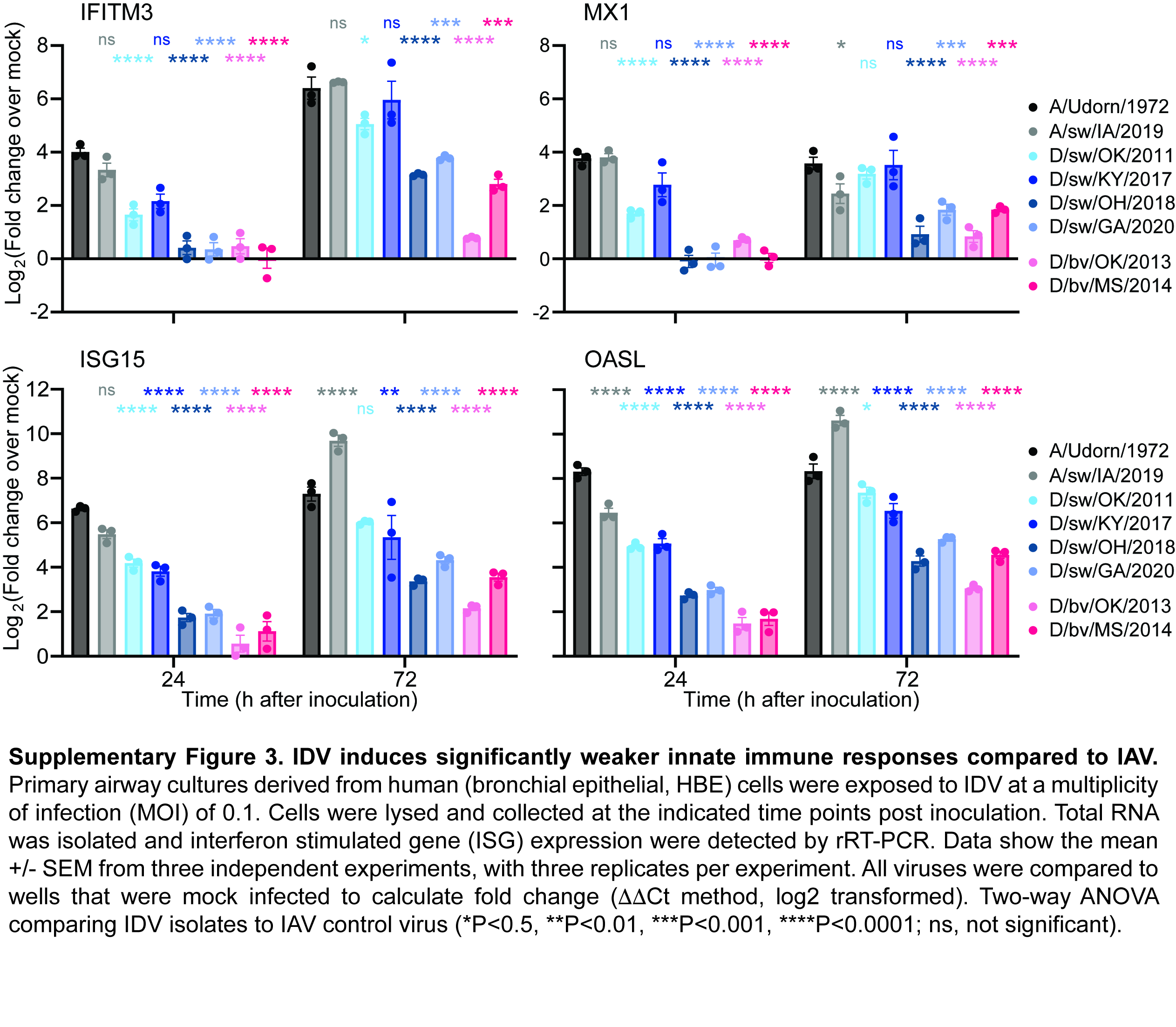

Supplement: Supplement 2 [file media-2.tif]

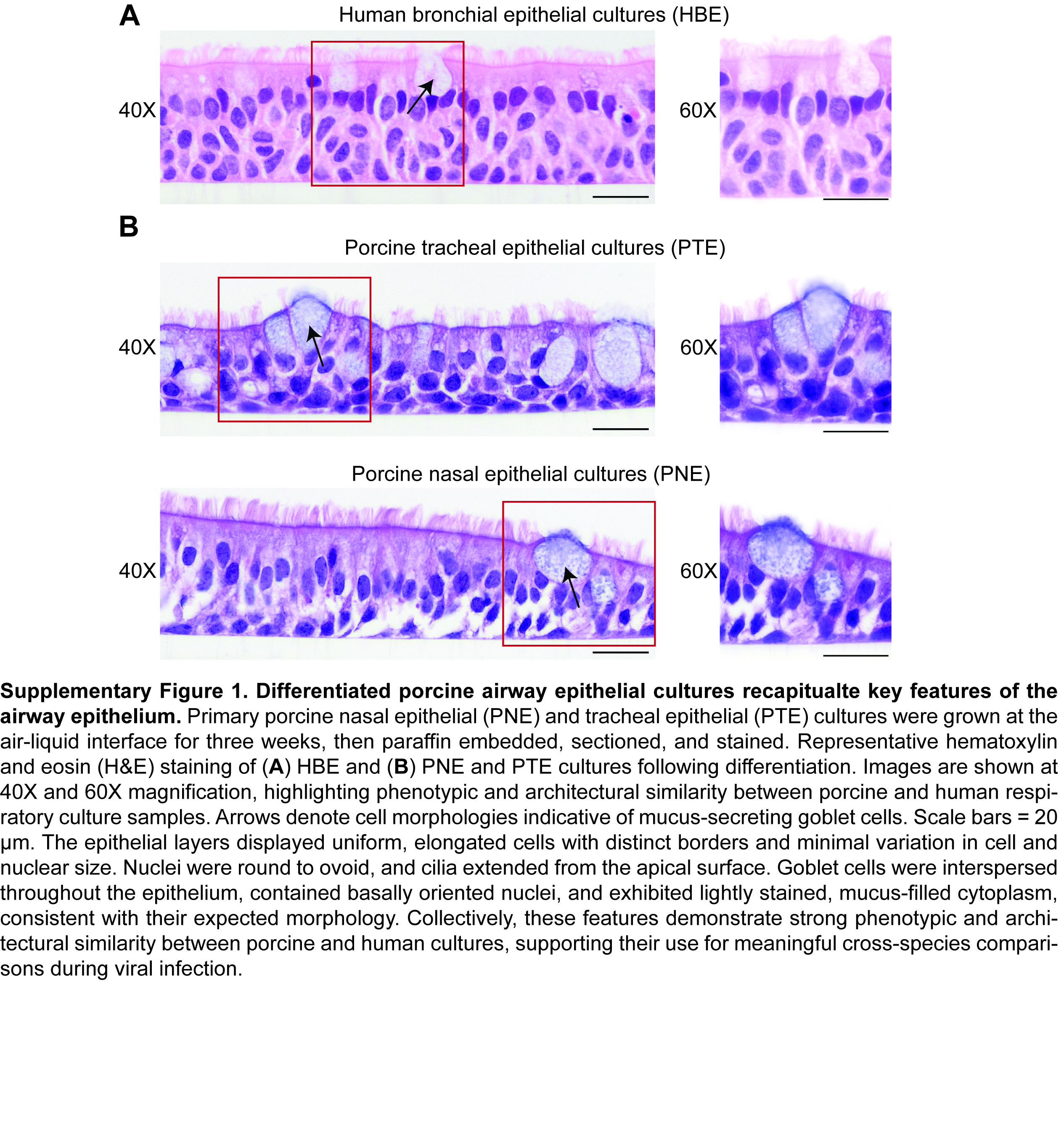

Supplement: Supplement 3 [file media-3.tif]
