# Supplementary material for: Efficient replication of influenza D virus in the human airway underscores zoonotic potential
Source: bioRxiv. 2026 Feb 8:2026.02.07.704474. Preprint. [Version 1] doi: 10.64898/2026.02.07.704474 (PMC12889682; doi:10.64898/2026.02.07.704474)
Supplement: Supplement 4 [file media-4.pdf]

Supplementary Table 1. Active influenza A surveillance organized by state and year.

	2017	2018	2019	2020	Total
Arizona		399			399
Colorado			400		400
Georgia		225	395	396	1016
Illinois	399	299	200		898
Indiana	1445	932	905	294	3576
Iowa	799	625	599	1600	3623
Kentucky	759	1309	200	250	2518
Michigan	796	281	324		1401
Mississippi			115		115
Ohio	2854	1973	1948	1602	8377
Oklahoma			900	198	1098
Texas			599		599
West Virginia	20	20			40
Total*	7072	6063	6585	4340	24060

*Numbers indicate total nasal swabs and snout wipes collected by state and year.
