# Supplementary material for: Efficient replication of influenza D virus in the human airway underscores zoonotic potential
Source: bioRxiv. 2026 Feb 8:2026.02.07.704474. Preprint. [Version 1] doi: 10.64898/2026.02.07.704474 (PMC12889682; doi:10.64898/2026.02.07.704474)
Supplement: Supplement 5 [file media-5.pdf]

Supplementary Table 2. Primer sequences used in this study.

Target	Forward primer (5′ - 3′)	Reverse primer (5′ - 3′)
IDV	TGGATGGAGAGTGCTGCTTC	GCCAATGCTTCCTCCCTGTA
IFNλ1	GGGACCTGAGGCTTCTCC	CCAGGACCTTCAGCGTCA
IFITM3	TTCGCCTACTCCGTGAAGTC	ATCCATAGGCCTGGAAGATCAG
MX1	TATGTGGGTTCCTGGCATCG	AAAGCCTGGCAGCTCTCTAC
ISG15	CAGCGAACTCATCTTTGCCAG	GGACACCTGGAATTCGTTGC
OASL	CCAGCAGTATGTGAAAGCC	AGCCTTCGTCCAACATGA
HPRT1	CATTATGCTGAGGATTTGGAAAGG	CTTGAGCACACAGAGGGCTACA
